# Seasonal changes in bone mineral parameters and body composition in youth football players

**DOI:** 10.1186/s13102-026-01724-7

**Published:** 2026-05-07

**Authors:** Antonio Hernandez-Martin, Javier Sanchez-Sanchez

**Affiliations:** 1 Department of Sports Sciences, Faculty of Medicine, Health and Sports, Universidad Europea de Madrid, Villaviciosa de Odón, Madrid, Spain; 2 Real Madrid Graduate School, Faculty of Medicine, Health and Sports, Universidad Europea de Madrid, Madrid, Spain

**Keywords:** Bone mineral density, Body composition, Youth football players, Seasonal changes, DXA assessment

## Abstract

This study aimed to examine seasonal changes in bone mineral characteristics and body composition among youth football players throughout a full competitive season. A total of 60 male participants were recruited and stratified into four competitive age categories (U10, U12, U14, U16). Assessments were conducted at three key timepoints: the beginning (P1), midpoint (P2), and end (P3) of the season. Bone Mineral Content (BMC), Bone Mineral Density (BMD), lean mass, and fat mass were measured using standardized dual-energy X-ray absorptiometry (DXA) procedures. Significant increases in whole-body BMC and lower limb BMC were observed at P3 compared to P1 and P2 (*p* < 0.01). Whole-body BMD also exhibited a significant rise at P3 relative to earlier measurements (*p* < 0.01). Additionally, a significant increase in the percentage of lean mass was detected from P2 to P3 (*p* < 0.05), accompanied by a general trend of fat mass reduction over time in all the age categories. The most pronounced and rapid improvements in bone parameters were observed in the older age categories (U14 and U16), particularly in whole-body BMC and BMD. Nonetheless, positive adaptations in bone health and body composition were evident across all age groups in football players by the end of the season. These findings describe the physiological adaptations observed during a competitive season, highlighting the musculoskeletal development that occurs in youth players during these key stages of growth.

## Introduction

Promoting bone health during childhood and adolescence is a major public health priority, as these stages are critical for the prevention of chronic bone-related conditions such as osteoporosis later in life. Osteoporosis is characterized by low bone mineral density (BMD), reduced bone mineral content (BMC), and deterioration of bone microarchitecture, leading to a heightened risk of fractures and significant associated healthcare costs [1, 2].

Up to 90% of peak bone mass is accrued before the age of 20, with adolescence being a key period for mineral accumulation [3]. It is estimated that 26% of adult BMC is acquired during adolescence [4], and that a 10% increase in BMC at this stage may reduce future osteoporotic fracture risk by up to 50% [5]. Between the ages of 10 and 16, boys accumulate approximately 39% of total adult BMC, 43% of lumbar spine BMC, 46% of total hip BMC, and 33% of femoral neck BMC [6].

Bone development is influenced by a complex interaction of genetic, hormonal, nutritional, and environmental factors, among which physical activity plays a central role [7, 8]. However, not all types of physical activity have the same effect on bone tissue. High-impact and weight-bearing activities are particularly effective in promoting bone mass gains due to the mechanical loads they impose [9]. While the World Health Organization recommends at least 60 min of moderate-to-vigorous physical activity per day for children, including bone-strengthening activities three times per week, the guidelines do not specify which sports may be most beneficial for bone development [10].

Several studies have shown that sports involving frequent mechanical loading, such as jumping, sprinting, and rapid directional changes, are more beneficial for bone health than low-impact or non-weight-bearing disciplines [11, 12]. Football, a multidirectional high-impact sport, meets these criteria and has been associated with greater BMD and BMC in children and adolescents compared to sedentary peers or those participating in less osteogenic sports [13, 14]. The biomechanical demands of football — including rapid acceleration, deceleration, impacts, and changes of direction — provide an ideal stimulus for bone adaptation during growth [15].

To accurately monitor these adaptations, it is essential to evaluate specific metrics: Bone Mineral Content (BMC), which measures the absolute amount of mineral in the bone (g), and Bone Mineral Density (BMD), which reflects the mineral concentration within the bone matrix (g/cm²) [13, 14]. In football, these metrics are crucial indicators of how the skeleton responds to high ground reaction forces and unilateral loading [10]. Furthermore, body composition parameters, specifically lean mass and fat mass, serve as vital markers of an athlete’s physical maturation and training efficiency [15]. While lean mass provides the internal mechanical tension necessary for osteogenesis through muscle-bone interaction, the optimization of fat mass is directly linked to metabolic health and athletic performance in youth players [16].

Despite these benefits, most of the existing literature is cross-sectional, and there is a lack of longitudinal research assessing how bone and body composition parameters evolve over a full competitive football season across different stages of biological development. This limits our understanding of when peak adaptations occur and how they might be optimized through age-appropriate training interventions.

Therefore, the aim of this study was to seasonal changes in bone mineral characteristics and body composition among youth football players throughout a full competitive season, in order to identify the periods of most pronounced bone-related adaptation. It was hypothesized that a competitive football season would be associated with significant increases in bone mineral parameters (BMC and BMD) and lean mass, alongside a reduction in fat mass across all age categories. Furthermore, these musculoskeletal adaptations were expected to be more pronounced and rapid in the older players (U14 and U16), potentially due to higher mechanical loading and greater training volume associated with these categories. The findings are expected to support the design of age-appropriate training interventions that optimize bone health and body composition throughout development, contributing to long-term musculoskeletal health in youth athletes.

## Materials and methods

### Design of the study

Data collection was performed at three predefined timepoints during the competitive season: baseline (P1, season start), mid-season (P2, approximately 4 months after P1), and end of season (P3, approximately 9 months after P1).

### Participants

A total of 60 male youth football players aged 9 to 16 years were recruited from a football academy in Toledo, Spain. Participation was voluntary, and athletes were grouped according to competitive category: U10, U12, U14, and U16. The inclusion criteria were attending more than 90% of training sessions, having a minimum of 2 years’ experience playing football and completing the 3 body composition measurements throughout the season. Being in one of the four categories analysed. Exclusion criteria were attending fewer than 90% of training sessions, failing to attend all three body composition measurements throughout the season, having less than two years’ experience playing football, and being in age groups below or above those analysed in the study. Descriptive characteristics of each age group are presented in Table 1.

**Table 1:** Descriptive characteristics of four groups of academic soccer

Measurement		U10	U12	U14	U16
		(*n* = 13)	(*n* = 13)	(*n* = 17)	(*n* = 12)
Age (years)	P1	9.6 ± 0.7bc	11.8 ± 0.8bc	13.6 ± 0.7c	15.8 ± 0.7
	P2	9.8 ± 0.8bc	11.3 ± 0.7bc	13.8 ± 0.8c	15.6 ± 0.5
	P3	10.6 ± 0.8bc	12.2 ± 0.6bc	14.5 ± 0.7c	16.4 ± 0.7
Height (cm)	P1	137.2 ± 18.1bc	143.7 ± 19.6bc	156.1 ± 36.7	170.3 ± 25.7
	P2	138.7 ± 21.3bc	145.6 ± 19.4bc	159.2 ± 33.3	171.1 ± 25.2
	P3	140.2 ± 25.1bc	147.3 ± 23.8bc	162.3 ± 32.8	172.4 ± 25.3
Weight (kg)	P1	31.5 ± 12.7bc	37.3 ± 31.2bc	46.9 ± 27.5	61.7 ± 43.6
	P2	32.7 ± 14.2bc	38.8 ± 31.8bc	48.1 ± 29.6	64.0 ± 44.7
	P3	32.2 ± 12.3bc	39.7 ± 32.6bc	50.8 ± 31.7	64.2 ± 44.6
Body mass index	P1	16.4 ± 1.6bc	18.2 ± 2.3c	18.8 ± 1.8c	21.1 ± 2.6
(BMI, kg · m^− 2^)	P2	16.7 ± 1.7bc	18.3 ± 2.4c	19.1 ± 1.9c	21.9 ± 2.5
	P3	16.2 ± 1.7bc	18.1 ± 2.5c	19.1 ± 1.7c	21.6 ± 2.7

(Mean ± SD; cm=centimeters; kg=kilograms; m=meters. P1 = period 1; P2 = period 2; P3 = period 3

Differences concerning the mentioned group at a (U12), b (U14), c (U16) *P* < 0.05

Differences with respect to the mentioned time in * (period 2), # (period 3) *P* < 0.05

Written informed consent was obtained from all participants and their legal guardians. The study protocol was approved by the Clinical Research Ethics Committee of Castilla-La Mancha and complied with the latest revision of the Declaration of Helsinki (Ref.: 489/24022020).

### Anthropometric assessments

Height and body mass were measured at all three timepoints (P1, P2, and P3). Body mass (kg) was recorded using an electronic scale (Seca 813, GmbH & Co, Hamburg, Germany), and height (cm) was measured using a portable stadiometer (Seca 214 GmbH & Co, Hamburg, Germany). Body mass index (BMI) was subsequently calculated as weight in kilograms divided by the square of height in meters (kg·m⁻²).

### Body composition and bone measurements

Body composition and bone parameters were assessed using dual-energy X-ray absorptiometry (DXA; Hologic Series Discovery QDR, APEX System Software Version 3.3.2, Bedford, MA, USA). The following variables were measured: bone mineral content (BMC, g), bone mineral density (BMD, g·cm⁻²), fat mass (g and %), and lean mass (g and %). All scans were performed in accordance with the manufacturer’s standardised protocols. Protocol for whole-body analysis: The participant lies on their back, with their arms at their sides (slightly away from the torso) and their feet positioned so that their legs do not touch, to avoid errors in the measurement of soft tissue and bone mass. Protocol for lumbar spine analysis: The patient lies in the centre of the table in a supine position. The lower limbs must be flexed at a right angle (90 degrees) at the hip and knee joints, usually with the legs supported on a positioning device or a foam block to flatten the lumbar lordosis and align the spine parallel to the table. Protocol for the hip (femur): A special positioning device is used to internally rotate the hip, ensuring that the femoral neck is parallel to the table.

The DXA device was calibrated daily using a lumbar spine phantom, and all assessments were conducted by the same trained technician to ensure procedural consistency. The anatomical regions analyzed included whole body (total, arms, trunk, and legs) and proximal femur (Ward’s triangle).

### Statistical analysis

Descriptive statistics are presented as means ± standard deviations. The total sample (*n* = 60) was stratified into four competitive categories: U10 (*n* = 13), U12 (*n* = 13), U14 (*n* = 17), and U16 (*n* = 12). Normality and homogeneity of variances were confirmed using the Kolmogorov–Smirnov and Levene’s tests, respectively (*p* > 0.05). A two-way mixed analysis of variance (ANOVA) was employed to evaluate the interaction between age category (between-subject factor: U10, U12, U14, U16) and seasonal period (within-subject factor: P1, P2, P3). To ensure the validity of the repeated measures analysis, Mauchly’s test of sphericity was performed. In instances where the assumption of sphericity was violated (*p* < 0.05), the Greenhouse–Geisser correction was applied to adjust the degrees of freedom. Bonferroni-adjusted post hoc tests were applied for pairwise comparisons. A post-hoc power analysis was conducted based on the sample size and experimental design; the study achieved a statistical power (1 − β) greater than 0.80 to detect a medium effect size (f = 0.25) at a significance level of α = 0.05. Effect sizes (ES) were calculated using Cohen’s d and interpreted as follows: trivial (< 0.19), small (0.20–0.49), medium (0.50–0.79), and large (> 0.80). The level of statistical significance was set at *p* < 0.05. All statistical analyses were conducted using SPSS software (v24.0; IBM Corp., Armonk, NY, USA), and graphical representations were generated using STATA (v16.0; StataCorp LP, College Station, TX, USA). 

## Results

**Fig. 1:**
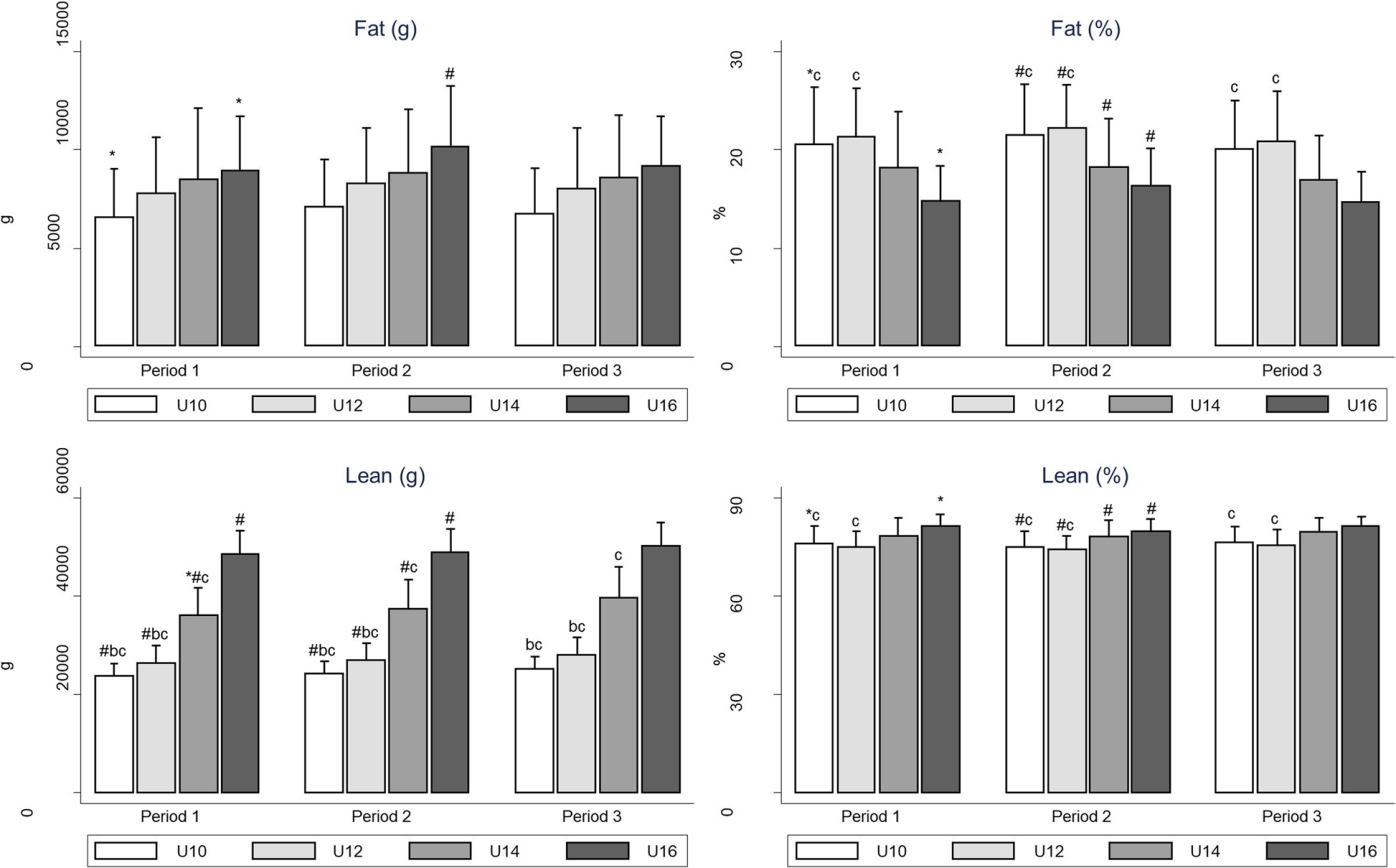
Body composition characteristics of young football players at three points in time throughout the season. Notes: Differences concerning the mentioned group at a (U12), b (U14), c (U16) *P* < 0.05. Differences with respect to the mentioned time in * (period 2), # (period 3) *P* < 0.05. g: grams; % percentage

Significant temporal changes were observed across the season in BMC (Table 2), BMD (Table 3), and body composition parameters (Fig. 1), with more pronounced improvements detected in the older age categories (U14 and U16).

**Table 2:** Bone mineral content characteristics of young football players at three points in time throughout the season

	Period 1				Period 2				Period 3			
	U10	U12	U14	U16	U10	U12	U14	U16	U10	U12	U14	U16
Whole body (g)	1081.55 ± 131.38#bc	1255.61 ± 143.06#bc	1568.93 ± 203.48*#c	2194.39 ± 243.27*#	1093.75 ± 132.73#bc	1265.75 ± 159.06#bc	1632.32 ± 213.55#c	2272.23 ± 228.12#	1129.33 ± 136.31bc	1323.68 ± 168.73bc	1749.42 ± 237.09c	2321.57 ± 216.30
L. Arm (g)	52.32 ± 8.78bc	63.22 ± 9.91bc	80.88 ± 15.22*#c	127.47 ± 20.89*	52.62 ± 8.34bc	63.65 ± 11.12bc	86.79 ± 19.02#c	132.40 ± 20.14	53.87 ± 9.53bc	68.26 ± 12.17bc	103.02 ± 35.99c	137.57 ± 20.78
R. Arm	54.02 ± 9.83bc	63.45 ± 9.48c	93.81 ± 50.29c	136.94 ± 19.60	52.59 ± 7.76bc	62.28 ± 9.70bc	87.93 ± 16.06#c	141.93 ± 22.12#	54.41 ± 9.26bc	65.42 ± 10.48bc	95.07 ± 19.59c	146.01 ± 20.52
Trunk (g)	269.70 ± 32.61bc	303.98 ± 42.41bc	421.80 ± 73.82*#c	627.86 ± 76.83*#	267.35 ± 35.27bc	310.92 ± 45.94bc	443.10 ± 90.86#c	661.88 ± 73.37	275.38 ± 35.61bc	321.39 ± 47.90bc	471.91 ± 89.84c	666.88 ± 70.49
L. Leg (g)	195.42 ± 32.27#bc	240.12 ± 33.97#bc	325.73 ± 48.84*#c	461.92 ± 66.12#	204.23 ± 30.67#bc	247.58 ± 33.66#bc	343.15 ± 50.32#c	466.92 ± 55.23#	214.06 ± 34.35bc	259.56 ± 38.55bc	370.58 ± 59.34c	476.78 ± 52.47
R. Leg (g)	198.22 ± 29.12#bc	236.23 ± 32.43#bc	315.84 ± 44.85*#c	435.80 ± 53.21*#	205.33 ± 30.09#bc	244.49 ± 39.00#bc	335.92 ± 49.74#c	452.17 ± 52.83#	214.06 ± 31.98bc	258.37 ± 41.58bc	360.85 ± 58.90c	463.46 ± 45.99
Ward’s triangle (g)	0.82 ± 0.12*#c	0.85 ± 0.10c	0.93 ± 0.15#c	1.10 ± 0.13#	0.89 ± 0.15c	0.91 ± 0.05c	0.96 ± 0.15#c	1.12 ± 0.10#	0.91 ± 0.15c	0.93 ± 0.06c	1.02 ± 0.13c	1.17 ± 0.15

*BMC* bone mineral content, *g* grams, *L* left, *R* right

Differences concerning the mentioned group at a (U12), b (U14), c (U16) *P* < 0.05

Differences with respect to the mentioned time in * (period 2), # (period 3) *P* < 0.05

**Table 3:** Bone mineral density characteristics of young football players at three points in time throughout the season

	Period 1				Period 2				Period 3			
	U10	U12	U14	U16	U10	U12	U14	U16	U10	U12	U14	U16
Whole Body (g/cm^2^)	0.81 ± 0.04#abc	0.88 ± 0.06#c	0.94 ± 0.06*#c	1.10 ± 0.07*#	0.82 ± 0.05#bc	0.88 ± 0.06#bc	0.96 ± 0.06#c	1.12 ± 0.07#	0.83 ± 0.05abc	0.90 ± 0.06bc	0.99 ± 0.06c	1.14 ± 0.07
L. Arm (g/cm^2^)	0.54 ± 0.04bc	0.57 ± 0.05bc	0.63 ± 0.05#c	0.74 ± 0.05	0.53 ± 0.04bc	0.57 ± 0.05bc	0.63 ± 0.04#c	0.73 ± 0.05	0.53 ± 0.05bc	0.058 ± 0.05bc	0.67 ± 0.08c	0.74 ± 0.05
R. Arm (g/cm^2^)	0.53 ± 0.05bc	0.56 ± 0.04bc	0.66 ± 0.12c	0.75 ± 0.04	0.53 ± 0.04#bc	0.55 ± 0.05#bc	0.64 ± 0.04c	0.75 ± 0.04#	0.54 ± 0.03bc	0.57 ± 0.04bc	0.65 ± 0.04c	0.77 ± 0.05
L. Leg (g/cm^2^)	0.81 ± 0.06#abc	0.90 ± 0.07#bc	1.01 ± 0.08*#c	1.23 ± 0.10#	0.82 ± 0.06#abc	0.91 ± 0.09#bc	1.04 ± 0.07#c	1.25 ± 0.10#	0.84 ± 0.06abc	0.93 ± 0.08bc	1.08 ± 0.08c	1.27 ± 0.10
R. Leg (g/cm^2^)	0.80 ± 0.05*#abc	0.90 ± 0.07bc	1.00 ± 0.06*#c	1.23 ± 0.09*#	0.82 ± 0.06bc	0.90 ± 0.09bc	1.05 ± 0.08#c	1.25 ± 0.09#	0.83 ± 0.05abc	0.92 ± 0.09bc	1.08 ± 0.08c	1.28 ± 0.09
Triangle Wards (g/cm^2^)	0.73 ± 0.10*#c	0.75 ± 0.06#c	0.80 ± 0.12#c	0.94 ± 0.10#	0.77 ± 0.14c	0.78 ± 0.05c	0.82 ± 0.12#c	0.96 ± 0.10#	0.78 ± 0.13c	0.81 ± 0.05c	0.86 ± 0.10c	0.99 ± 0.12

*BMC* bone mineral content, *BMD* bone mineral density, *g/cm2* grams / centimetre squared, *L* left, *R* right

Differences concerning the mentioned group at a (U12), b (U14), c (U16) P < 0.05

Differences with respect to the mentioned time in * (period 2), # (period 3) P < 0.05

Across all age groups, whole-body BMC and leg BMC (both right and left) increased significantly from the beginning (P1) to the end (P3) of the season (*p* < 0.01; ES: 0.48–1.54). These changes were particularly marked in U14 and U16 players. In the upper limbs, left arm BMC increased significantly in U14 players at both P2 and P3 compared to P1 (*p* < 0.01; ES: 0.34–0.43), while U16 players showed significant gains only at P2 (*p* < 0.01; ES: 0.24). Right arm BMC increased from P2 to P3 in both U14 and U16 players (*p* < 0.01; ES: 0.23–0.36). Trunk BMC rose significantly from P1 to P2 and again at P3 in U14 and U16 players (*p* < 0.001), with the greatest change between P2 and P3 observed in U14 (*p* < 0.01; ES: 0.31). In Ward’s triangle, significant BMC increases from P1 to P3 were noted in U10, U14, and U16 players (*p* < 0.05), with U14 and U16 also showing gains from P2 to P3 (*p* < 0.01; ES: 0.40–0.42).

Regarding BMD, whole-body values increased significantly at P3 compared to P1 and P2 in all categories (*p* < 0.01; ES: 0.35–1.47). U14 and U16 players also showed significant increases at P2 compared to P1 (*p* = 0.04). BMD in the left arm improved significantly only in U14 players at P3 versus P1 and P2 (*p* < 0.05; ES: 0.61–0.66). In the right arm, significant gains from P2 to P3 were detected in U10, U12, and U16 players (*p* < 0.05; ES: 0.58–1.39). Lower limb BMD increased across all categories in the left leg at P3 compared to both previous timepoints (*p* < 0.05; ES: 0.29–1.74), with additional increases at P2 in U14 players (*p* = 0.08). Right leg BMD improved significantly in U14 and U16 players across all three timepoints, with the largest differences occurring between P2 and P3 (*p* < 0.01; ES: up to 2.22). In U10 players, an increase in right leg BMD at P3 compared to P1 approached significance (*p* = 0.09; ES: 1.00). BMD in Ward’s triangle increased in all age groups at P3 compared to P1 (*p* < 0.05), with U14 and U16 players also showing gains from P2 to P3 (*p* < 0.05; ES: 0.37–1.63).

Consistent age-related differences were also observed across all periods. U14 and U16 players exhibited significantly higher BMC and BMD in nearly all anatomical regions compared to U10 and U12 players (*p* < 0.01), with the greatest effect sizes in the legs, arms, and trunk (ES: up to 5.13). Whole-body BMD was consistently highest in U16 players throughout the season (*p* < 0.01), followed by U14, U12, and U10, respectively. Notably, U16 players demonstrated significantly higher BMD values in both arms, legs, and Ward’s triangle at every measurement point compared to all younger categories.

Body composition outcomes revealed similar temporal improvements. Absolute lean mass increased significantly from P2 to P3 in all age groups (*p* < 0.01; ES: 0.23–1.09), with a marked rise in U14 players already evident at P2 (*p* < 0.01; ES: 0.22). The percentage of lean mass also improved in all groups at P3 relative to P2 (*p* < 0.05). Fat mass showed a more complex pattern: U10 and U16 players experienced an increase from P1 to P2 (*p* < 0.05), followed by a significant decrease at P3 in the U16 group (*p* < 0.01). Fat mass percentage rose at P2 in U10 and U16 players (*p* < 0.05) but then declined significantly across all categories at P3 (*p* < 0.01; ES: 0.21–1.12).

Between-category comparisons confirmed that U14 and U16 players consistently had greater lean mass (both absolute and relative) compared to U10 and U12 players at all timepoints (*p* < 0.01; ES: 0.21–1.32). In contrast, U10 and U12 players showed significantly higher fat mass percentages than U16 players throughout the season (*p* < 0.05; ES: 0.30–1.63). 

## Discussion

The primary aim of this study was to analyze longitudinal changes in BMC, BMD, fat mass, and lean mass across different anatomical regions in youth football players aged 9 to 16 years, over the course of a full competitive season. The findings revealed significant improvements in all body composition and bone-related variables observed throughout the nine-month period coinciding with the football season, with more rapid and pronounced adaptations observed in U14 and U16 players.

In terms of body composition, all age groups demonstrated an increase in lean mass and a reduction in fat mass by the end of the season. These results are consistent with prior longitudinal studies, such as those by Zouch et al. [13], which reported increases in both fat mass and lean mass after five months of football training in 10–13-year-old players. However, the present study identified a further reduction in fat mass (4–9%) and increase in lean mass (6–11%) at nine months across all groups, reflecting the progressive physiological changes that take place throughout the full seasonal cycle. Similar trends have been reported in interventional studies comparing football-trained children with age-matched controls, where football participation was associated with significant improvements in lean mass and reductions in fat percentage [14–16]. The significant increase in absolute lean body mass observed across all age groups is particularly relevant, as it may have served as a key factor in the improvement of the bone health parameters identified in this study. The internal stress exerted by developing muscle mass on bone, the mechanostat, acts as a key stimulus for mineral accumulation, suggesting that the seasonal increases in BMD and BMD were closely related to these concurrent improvements in lean body mass [14].

Contrary to Zouch et al.’s mid-season findings [13], the current data showed a transient increase in fat mass at mid-season in some groups, which may be related to the holiday period and temporary reductions in physical activity. Nevertheless, by the season’s end, all categories demonstrated improved body composition values. These fluctuations suggest a relationship between consistent athletic participation and the physiological adaptations observed over the course of a full competitive season.

With regard to bone parameters, the most remarkable observations were noted in BMC and BMD across the whole body and lower limbs. Throughout the nine-month period coinciding with the competitive season, players across all age categories exhibited significant improvements in these variables. Notably, older players (U14 and U16) displayed early adaptations, with measurable gains already evident at the four-month mark. For example, U16 players increased whole-body BMC by approximately 3.5% and BMD by 1.8% from baseline to mid-season, reaching improvements of 5.8% and 3.6%, respectively, by the end of the season. These findings describe the progressive and cumulative changes in bone mass and density observed during late adolescence in youth footballers. While the pattern of these adaptations aligns with the specific mechanical demands of the sport, it is important to recognize that these changes occur alongside natural processes of biological maturation and rapid growth characteristic of these developmental stages.

These results are aligned with those of Vicente-Rodríguez et al. [17], who showed that short-term football training was insufficient to elicit significant BMC gains in prepubescent boys. Similarly, other studies comparing football players with moderately active controls (engaging in ~ 64 min/day of moderate-to-vigorous physical activity) found no significant differences in BMC or BMD after 12 months, which suggests that factors such as training load and intensity are key elements associated with the bone adaptations observed during the seasonal cycle [18, 19].

Importantly, the more advanced changes seen in U14 and U16 players could be related to several factors: (1) higher baseline body mass generating greater mechanical load on bone [20], (2) increased training frequency and volume [21], and (3) greater cumulative years of sport participation. López-García et al. [22] also observed similar bone gains in 15-year-old footballers after only five months of training, reinforcing the idea that older youth may respond more quickly to mechanical stimuli. However, it is essential to emphasize that these changes occur during peak stages of biological growth and maturation. Moreover, consistent participation over at least three years has been shown to significantly enhance whole-body BMC in adolescent footballers when compared with untrained peers [23].

Lower limb BMD, particularly in the legs, improved across all groups, likely due to the osteogenic nature of football-specific actions (e.g., sprinting, jumping, tackling), which predominantly engage the lower extremities [24]. While upper limb changes were less marked, modest increases were noted in U14 and U16 players, potentially reflecting secondary transfer effects of whole-body training.

Furthermore, improvements in Ward’s triangle BMD—an indicator of trabecular bone integrity—were recorded in all age groups by the end of the season. These findings echo those from studies in U13 and U15 footballers, where increases in Ward’s triangle BMD were observed after 5 to 12 months of sport-specific training [22, 25]. While these results describe a positive trend in bone quality throughout the competitive season, they likely reflect the combined influence of sport-specific loading and the natural processes of skeletal development characteristic of these age groups.

The present findings are also consistent with recent meta-analytic evidence demonstrating that football is associated with greater osteogenic responses than other sports such as swimming or cycling, particularly in prepubertal and pubertal stages [26]. These observations are frequently linked to the repeated, multi-directional mechanical loads inherent in football, which are thought to favor periosteal bone formation and cortical thickening during growth [27]. The sport’s combination of high ground reaction forces, unilateral loading, and rapid changes of direction represents a specific mechanical environment that coincides with the significant bone adaptations observed during these critical growth windows.

This study has certain limitations that should be acknowledged. Firstly, dietary intake, including key nutrients such as calcium and vitamin D, was not monitored, which specifically affects the interpretation of the observed mineral accumulation. Given that these nutrients are essential for bone mineralization during growth, the absence of this data prevents us from determining whether the significant increases in whole-body BMC and BMD were enhanced by optimal nutritional status or whether, conversely, potential deficiencies limited the magnitude of skeletal adaptations. Secondly, physical activity outside the club’s structured training sessions was not accounted for. This introduces a confounding variable in the interpretation of total mechanical load; it is possible that participants with additional high-impact activities experienced a higher cumulative osteogenic load, which could lead to an overestimation of the effect attributed exclusively to the football training programmed. Third, biological maturation was not directly assessed. Given that participants were aged 9 to 16 years, they were navigating critical windows of pubertal growth and rapid mineral accrual. Without this maturation data or a non-active control group, it is difficult to fully isolate the specific effects of football from natural biological development. Consequently, these findings should be interpreted as seasonal physiological adaptations occurring concurrently with growth. Nevertheless, the fact that the most robust and consistent adaptations were localized in the lower limbs and Ward’s triangle, regions that bear the specific loads of football, such as sprints and jumps, suggests that the mechanical stimulus of the sport was the predominant determining factor over other external or systemic stimuli.

## Conclusion

In summary, significant increases in bone mineral content, bone mineral density, and lean mass, along with a reduction in fat percentage, were observed in youth football players throughout a nine-month competitive season. The most pronounced and early physiological changes were identified in the U14 and U16 categories, where improvements in bone parameters were already detectable by mid-season. While these findings occur concurrently with natural processes of biological maturation and growth, the localized nature of the adaptations, particularly the robust gains in the lower limbs and Ward’s triangle, highlights the musculoskeletal development that characterizes youth athletes during a seasonal cycle. These results provide valuable descriptive evidence regarding the seasonal dynamics of bone health and body composition in young footballers.

### Practical application

Below is a series of practical applications for coaches and professionals:

- Prioritize Season Long-Consistency: Encourage high attendance rates (exceeding 90%) throughout the full nine-month cycle, as musculoskeletal adaptations are progressive and cumulative.
- Specific Drills for Osteogenesis: Incorporate training sessions that emphasize high-impact, multi-directional actions, such as rapid changes of direction, sprints, and jumps, which provide the necessary mechanical stimuli for bone thickening and mineral accrual.
- Monitor Older Categories Closely: Given that U14 and U16 players show earlier and more robust adaptations, training loads should be carefully managed in these groups to maximize their greater responsiveness to osteogenic stimuli.
- Mitigate Holiday Detraining: Implement maintenance programs during holiday or mid-season breaks to prevent the transient increases in fat mass and temporary reductions in physical activity observed at the season’s midpoint.
- Balanced Development: While football naturally favors lower limb development, professionals should include complementary trunk and upper-body exercises to ensure balanced musculoskeletal health across all anatomical regions.

## Data Availability

The data supporting the findings of this study are available from the corresponding author upon reasonable request. Due to ethical and privacy concerns, the data cannot be made publicly available. Anonymized datasets can be shared with qualified researchers under a data use agreement approved by the institutional ethics committee.

## Abbreviations

U10: Under-10
U12: Under-12
U14: Under-14
U16: Under-16
P1: Timepoint 1
P2: Timepoint 2
P3: Timepoint 3
BMC: Bone Mineral Content
BMD: Bone Mineral Density
DXA: Dual-energy X-ray Absorptiometry
BMI: Body Mass Index
ANOVA: Analysis of Variance
ES: Effect Size
SPSS: Statistical Package for the Social Sciences
STATA: Data Analysis and Statistical Software
kg: Kilogram
cm: Centimeter
g: Gram
%: Percentage
